# Assessing tap water awareness: The development of an empirically-based framework

**DOI:** 10.1371/journal.pone.0259233

**Published:** 2021-10-29

**Authors:** Stijn Brouwer, Nicolien van Aalderen, Steven Hendrik Andreas Koop

**Affiliations:** 1 KWR Water Research Institute, Nieuwegein, The Netherlands; 2 Department of Sociology, University of Antwerp, Antwerp, Belgium; 3 Copernicus Institute of Sustainable Development Utrecht University, Utrecht, The Netherlands; University of Miami, UNITED STATES

## Abstract

Despite the often emphasized importance of water awareness, and notwithstanding the fact that calls for increasing public awareness are becoming commonplace, most studies do not define the concept, let alone operationalise it into measurable units. This is, however, essential to measure and evaluate efforts related to water awareness such as public campaigns, customer communication and behavioural interventions. To address this gap, we conceptualise, operationalise and assess tap water awareness, hereby differentiating between cognitive awareness (head), affectional awareness (heart), and behavioural awareness (hands). In parallel, we also differentiate between tap water quality, quantity and system. By building on a variety of contemporary conceptual insights in literature and a series of expert interviews, an assessment framework is developed. A cohesive set of nine awareness components are identified and operationalised into a set of tangible questions which are put to the test in a large-scale online survey (n = 1003) in the Netherlands, applying both a traditional and modern segmentation approach based on four types of perspectives (‘quality & health concerned’, ‘aware & committed’, ‘egalitarian & solidary’, and ‘down to earth & confident’). Based on the analysis of the results of the first empirical application of our tap water awareness assessment framework, we conclude that—with a score 53.5 points out of 100—tap water awareness in the Netherlands shows ample room for improvement. Interestingly, most significant variations in awareness are generally not related to sociodemographic factors but rather apply to the four customer perspectives on drinking water that are based on people’s subjective views and preferences.

## 1. Introduction

Global access to drinking water and the combined importance of the management of freshwater resources and access to drinking water and sanitation, identified as a Sustainable Development Goal (SDG6), have improved over the last decades [1, 2]. At the same time, many freshwater resources are shrinking irreversibly due to increasing water demands, large-scale pollution, seawater intrusion, and changing precipitation patterns and temperatures [3, 4]. Indeed, water scarcity is recognized as one of the most important global risks, both in terms of likelihood and impact [5]. In many world regions, including Europe, the projected rise in temperatures will bring drier soils and more frequent and severe heatwaves, likely leading to a sharp increase in the number of people living under water stress and causing severe damage to ecosystems and agricultural practices [6]. Perhaps less distressing, but no less important, is the fact that such drought episodes often lead to large peaks in water demand. Because of abrupt changes in pressure, such peaks occasionally result in tap water discolouration and require expensive infrastructure augmentation as well as high energy costs to treat, pump and maintain the water supply network [7, 8]. In addition to water quantity stress, also the quality of drinking water sources are increasingly under pressure. Beyond water scarcity, the emission of pesticides, biocides and nutrients from agriculture, the release of hazardous chemicals from households (e.g. pharmaceuticals and detergents) and from industrial processes, as well as the increasing number of emerging substances form a growing threat affecting water resources [9, 10]. Against this backdrop, public awareness about freshwater availability, environmental impact, the need for more sophisticated and more costly treatment processes, and consequently the necessity to use water more efficiently, now becomes more important than ever before. This urgency is for instance reflected in the influential work on water governance of the Organization for Economic Cooperation and Development (OECD), warning for a lack of public awareness in, for instance the Netherlands, with respect to too little, too much and too polluted water [11, 12].

Despite the often emphasized importance of water awareness, and notwithstanding the fact that calls for increasing public awareness are becoming commonplace, most studies, including previous work of the authors of this paper, do not unequivocally define the concept, let alone, operationalise it concurrently into measurable units [e.g. 13–17]. Awareness is often referred to in broad terms and used in an exchangeable fashion across different water-related elements and goals such as surface water improvements [18–21], drinking water quality [22, 23], and water quantity [14, 24]. The concept of awareness itself is multifaceted, and found to encompass different things, including, but certainly not limited to, cognitive knowledge, the degree of involvement and having a specific attitude. In this paper, we will focus on tap water awareness specifically, hereafter referred to as TWA. The key objective is to conceptualise, operationalise and assess TWA. To this end, we build on a variety of contemporary conceptual insights, develop an assessment framework, and empirically test and apply this framework in the context of the Netherlands. This last step, wherein we assess the TWA of drinking water customers, involves the division of respondents into different segments, both classical, based on ‘hard’ sociodemographic differences such as gender, age and educational background, as well as modern, based the more ‘soft’ differences on the basis of subjective views and perspectives [25]. In doing so, we also intend to further explore the value of perspective-based customer segmentation versus the more classical approaches of segmentation.

The remainder of this paper is organized into five sections. Section 2 presents the conceptual foundations of TWA, and distinguishes between cognitive, affectional and behavioural awareness of tap water. Along with an introduction to the four applied customer perspectives on drinking water, this study’s methodology, encompassing the development and implementation of a framework by |means of interviews and an online-survey questionnaire, is explained in section 3. Section 4 introduces the actual operationalisation of the assessment framework, allowing to calculate the TWA ‘profiles’ of individuals and segments of the population. In addition, it presents the results of the first empirical application of our TWA assessment framework in the Netherlands. Finally, section 5 provides a discussion and concluding remarks on our framework and the awareness results, as well as a reflection on the strengths and limitations of our approach.

## 2. The conceptualisation of tap water awareness: Head, heart & hands

The development of an empirically-based assessment framework for analysing TWA was primarily guided by a conceptual and theoretical reflection on water awareness, as well as an exploration of how this concept has been used and translated in everyday awareness campaigns. The underlying assumption in many of these campaigns is grounded in the premise that awareness is determined by cognitive knowledge, and that raising awareness is about increasing public understanding. Also in the literature, the cognitive narrative of TWA seems prevailing [21]. An example of such a cognitive focus is provided by Ntengwe [26: p.1303], who defines awareness of tap water customers as “a condition whereby customers know what it takes to produce water and have it delivered at the tap near or in households”.

Beyond a merely cognitive conceptualization of awareness, other scholars embrace a broader rationale by incorporating the notions of perception and attitude, hereby acknowledging the growing importance of subjective experience in the drinking water domain [25]. Such a more inclusive notion of awareness merges cognition with how this information is understood, interpreted and perceived. Since many studies, including Doria [27] and Doria [22], suggest that beyond traditional quality standards, more perceptive-oriented parameters such as taste, odour, colour and turbidity or trust in water utilities have become decisive in how people regard and trust the quality of their tap water, we regard this an important element too. Petrescu [14] is one of the studies that operationalised such a broader conceptualisation of awareness, and accordingly, also assessed people’s attitude towards the utility’s service delivery, water quality and price, next to people’s knowledge about, for instance, the water utility and water sources.

In their study of surface water awareness, Boer et al. [18] principally define awareness as the realisation of the existence of something. Moreover, along with cognition and affection they identified a third component of awareness: behavioural intention. Next to the physical and social-cultural environment, they argue that people’s cognition, affection and desired behaviour influences the way they act. As such, the effects of people’s actions can reinforce or lessen their awareness. Whereas Boer et al. [18] conceptualise water awareness merely as preceding and feeding behaviour, others regard behaviour as a pivotal element of awareness itself, for instance in the field of pro-environmental behaviour [28]. In this domain, the idea of environmentally conscious behaviour, which can be defined as human action motivated by a concern for the environment, is well established [29]. In the domain of water this more inclusive conceptualisation of awareness is for instance reflected in the work of Wang et al. [13]. In their review of public TWA in the Chinese province Hainan, they not only assessed people’s knowledge of contamination accidents (cognition), their degree of trust in drinking water safety (attitude), but also included behavioural questions, such as people’s use of bottled water.

Building on the work of these scholars, our conceptualisation of TWA is composed of three dimensions: (I) cognitive awareness (e.g. knowledge on the composition of tap water), (II) affectional awareness (e.g., the extent to which people take tap water for granted), and (III) behavioural awareness (e.g., a conscious use of tap water and the preventing spilling). This holistic three-dimensional approach is consistent with the organising principle of head (cognitive), heart (affective), and hands (behavioural). This head, heart & hands approach is not only a pivotal element in the Waldorf model of education [30], but has also been widely used in transformational learning theory [31], and holistic approaches on ecoliteracy and sustainability education [32, 33]. For instance, in the description of Orr [33] of how to approach education for sustainability, he suggests that the head, heart, and hands approach integrates intellect, emotion and body, whereas Singleton [31] uses this approach to illustrate how people may progress from knowing to caring, to loving, and to doing. The same head, heart & hands approach has also been applied in other fields, including research on engagement [34], and is consistent with the three dimensions of attitudes identified in social psychological research [35]. In parallel to this threefold conceptualisation of TWA, we propose an additional more practical classification that is based on substantive characteristics of drinking water: water quality, water quantity and water system.

From a substantive point of view, two rather separated strands can be distinguished from the literature: awareness about tap water quality and awareness about tap water quantity and consumption. Water quality literature focusses mainly on people’s knowledge or ignorance of water quality [23, 36, 37] and their risk perception [38]. Studies on awareness related to water quantity, instead, predominantly stem from conservation psychology, where behavioural aspects regarding the efficient use of energy and resources are studied. Here awareness is regarded as an element of conservative behaviour [14, 24, 39, 40]. A third, yet rather overlooked strand which we consider important in our conceptualisation, relates to awareness of tap water in its broader context: the water system. Do people know where their tap water originates from? To what extent do they understand the interrelatedness of the water cycle? Do they know the name of their water utility? Do they care, and if they care, to what degree is this knowledge translated into a more conscious behaviour? Petrescu [14] is one of the studies that already appreciated the importance of this water system element, and for that reason included questions on people’s knowledge about, for instance, their water utility and source of tap water. Accordingly, in this study we define TWA as the subjective reflection and practice about tap water in its broader context. Section 4 will further detail this conceptualisation, and introduce the actual operationalisation of TWA in a three-by-three dimensioned assessment framework. This framework is designed to assess individual and (specific) segment TWA profiles. The next section will first elaborate on the methodology that this study applies.

## 3. Methodology

The development of this study’s TWA assessment framework was primarily dictated by the theoretical considerations as outlined in the Section 2, which will be substantiated and applied in the following sections. This process was strengthened by the outcomes of three semi-structured face-to-face expert interviews (conducted in the summer of 2019) with professionals in the field of drinking water. The interviews were recorded, summarized and shared with the interviewees for review and approval. The interviewees consisted of one head of communications, one senior strategist and one programme manager working on the topic of TWA. The interviewees were informed about the study and had the opportunity to ask questions prior to the interview. Then, informed consent was received orally and formally recorded. Both the theoretical study and the expert interview analysis were used to define and to elaborate the conceptual categories (i.e., head, heart and hands) and substantive categories (i.e., water quality, water quantity and water system), as well as to formulate and specify survey questions, i.e. the operational questions of our TWA framework. Furthermore, three additional content experts (including one head communications, and two customer managers based at three different Dutch water utilities) generated items for inclusion in the questionnaire. In the final draft stage, the same experts modified and refined some questions, and helped to further improve the flow of the questionnaire and refine the weighing system. The questionnaire was pre-tested during seven individual interviews with a varied group customers of mixed gender, age and educational level (including one non-native speaker) to evaluate the clarity of each question and to assess the questionnaire’s comprehensiveness [41]. Based on their advice and suggestions, the questionnaire was revised to its final form.

The final instrument was an online-survey questionnaire consisting of 33 questions. The first section of the questionnaire asked for sociodemographic information including gender, age, residence, annual household income, and highest level of education attained. In order to elicit the respondents’ subjective views on drinking water, we built on the work Brouwer et al. [25] who in their modern segmentation approach distinguished four different customer perspectives on drinking water, summarized in Table 1. Accordingly, in order to determine to which perspective the respondents feel most connected to, we used their matrix question consisting of four sets of propositions, numbered A to D, and asked respondents which set of propositions best represented their individual perceptions.

**Table 1:** Customer perspectives.

Perspective	Description
**(I) Quality & health concerned**	Customers characterised by a focus on personal preferences and needs, especially regarding their own health and tap water quality
**(II) Aware & committed**	Customers characterized by pro-environmental values and collective sustainability ideals
**(III) Egalitarian & solidary**	Customers characterized by great sense of solidarity with less-favoured households, low-income countries, and future generations
**(IV) Down to earth & confident**	Customers characterized by a great confidence in the responsibility of drinking water utilities, along with the desire not to be bothered about drinking water

The second section of the questionnaire contained a mix of head (cognitive), heart (affective), and hands (behavioural) statements and questions on tap water quality, quantity and system issues, i.e. the operational questions of our TWA framework as listed in Table 3. The questionnaire contained mostly close-ended questions with limited response choices, such as nominal (yes/no) questions, multiple choice questions with four to five answers, or questions with ordinal responses (strongly disagree, somewhat disagree, neutral, somewhat agree or strongly agree). All survey questions were compulsory, with exception of the sociodemographic questions and, as to avoid incorrect answers, the question about the volume record on the respondents’ latest water bill.

To calculate the awareness profiles, the questionnaire was accompanied with an elaborate weighing system, further detailed in S1 Appendix, which we based on expert judgment and logical reasoning. For each of the three dimensions—head (cognitive), heart (affective) or hands (behavioural)–an equal sum of maximum of 36 points could be scored, whereby questions were awarded with between 2 and 8 points. Accordingly, one could score maximal 108 awareness points. The number of points per question was divided in such a way that the maximum score for all three components—water quality, water quantity, and water system—is also 36 points. No scores were assigned to the questions regarding sociodemographic information and the perspective matrix. The results of this study’s analysis are consistently reported as the percentages of point scored compared to the maximum score overall or the maximum score in each group.

A representative sample for the Netherlands was recruited through the panel of CasaGrande (CG) Research, an experienced market research agency, who also organized and coordinated the data collection. The survey was conducted in October 2019. The timing of the survey was aligned with the Dutch drinking water utilities. To avoid bias, a period of time was chosen when there were no foreseen major press releases on tap water issues or awareness raising campaigns. As part of the scoping process, CG Research implemented age, gender, educational, and regional quotas based on Dutch population census data. A priori, a minimum sample size of 1000 was specified. Only participants of age 15 years or older were selected, and respondents received a small monetary reward to participate. CG Research provided us with the final sample of n = 1003 respondents. All data were fully anonymized before access by the researchers. The demographic characteristics of the study sample are presented in Table 2. The results per question are presented in S2 Appendix.

**Table 2:** Study sample characteristics.

Variable	%
**Gender** (n = 1001)	
Women	54.4
Men	45.6
**Age** (n = 996) ^1^	
≤17	3.0
18–24	11.7
25–34	16.9
35–44	15.4
45–54	15.7
55–64	19.8
65≥	17.4
**Region** (n = 1003)	
Amsterdam, Rotterdam, The Hague and suburbs	17.2
Friesland, Groningen and Drenthe	8.7
North-Brabant, Limburg and Zeeland	24.1
North-Holland, South-Holland and Utrecht	26.2
Overijssel, Gelderland and Flevoland	23.7
**Education** (n = 1000)	
Low (primary school; lower secondary professional education; lower general secondary education)	23.9
Medium (intermediate vocational education; higher general secondary education; pre-university education)	39.2
High (bachelor, master, PhD)	36.9
**Perspective** (n = 999)	
Quality & health concerned	12.6
Aware & committed	32.7
Egalitarian & solidary	28.3
Down to earth & confident	26.4

^1^ The lower n for some categories reflects the fact that respondents were permitted to skip the sociodemographic question.

Based on the scoring methodology shown in Table 3, a score was assigned to each respondents’ answer. Next, the fraction of the maximum score was calculated and expressed as a percentage. For example, a 50% total awareness implies that a respondent has achieved half of the maximum attainable score. In this way, respondent’s scores could be clustered to scores of individual questions, components and dimensions. The total sample and each category (i.e., gender, age, education and customer perspectives) was repetitively tested for each question for normality (using the independent samples Kolmogorov-Smirnov test). In addition, Levene’s test was conducted to test for the assumption of the homogeneity of variance. The Kolmogorov-Smirnov test has not resulted in significant differences from normality. Levene’s test null hypothesis of homogeneity of variances was also not violated. Two-tailed ANOVA tests with planned contrasts have been conducted to test the null hypothesis that all groups are equal. An individual sub-group is consistently compared with the total of other sub-groups. For instance, the scores within the age category ≤17 years were compared with all other age categories. In addition, effect size (r) was calculated and interpreted according to Cohen [42] with r = 0.01 as very small effect (vs), r = 0.10 as small effect (s), r = 0.30 as medium effect (m), and with r = 0.50 as large effect (l) [43, 44]. Finally, the Benjamini-Hochberg procedure was performed for each null-hypothesis (i.e., each component and dimension) to decrease the false discovery rate. Hence, the statistical analysis enables an exploration of which categories have significantly higher or lower scores in total, with respect to dimensions and components.

**Table 3:** Tap water awareness framework.

Dimension	Component	Operational questions	Weighing (in points)	
**Cognition**	**(I) Water quality comprehension**	*My tap water contains… (none; a small quantity; or a large quantity of anthropogenic substances* * *)*	2	10
		*To your knowledge*, *is chlorine added to your tap water*?	4	
		*Are the quality requirements stricter for tap water or bottled water*?	4	
	**(II) Water consumption knowledge**	*Estimate the average daily water consumption of one person in the Netherlands*?	4	8
		*Estimate how much water*, *in litres*, *a conventional shower head uses per minute*?	4	
	**(III) Water system understanding**	*What is the source of your tap water*? *(surface water*, *ground water*, *etcetera)*	2	18
		*What is the name of your drinking water utility*?	4	
		*Which responsibilities do you think belong to the tasks of your drinking water utility*? *(swimming water management; sewage treatment; tap water purification etcetera)*	4	
		*Drinking water utilities are responsible for the quality of tap water*? *(up to the pumping station; the water meter; the tap)*	4	
		*What is the price for 1,000 litres (1 m*^*3*^*) of tap water*, *excluding taxes*?	4	
**Affection**	**(IV) Water quality perception**	*How safe do you perceive tap water in the Netherlands*?	8	16
		*In my view*, *clean tap water is something obvious*	4	
		*How often do you think about the quality of your tap water*?	4	
	**(V) Caring for water**	*I would like to save (more) tap water at home*	4	12
		*Every single day I experience 24 hours running tap water as special*	4	
		*How often do you think about your water consumption*?	4	
	**(VI) Sense of responsibility**	*I sometimes think about the origin of my tap water*	4	8
		*I feel a personal responsibility for protecting the quality of water in rivers*, *lakes*, *ditches and subsurface*	4	
**Behaviour**	**(VII) Quality -driven behaviour**	*In the past 24 months*, *have you ever actively looked for information on the quality and safety of Dutch tap water*? *(e*.*g*. *via the Internet or by contacting your drinking water utility)*	4	10
		*How often do you drink bottled non-sparkling water at home*?	6	
	**(VIII) Curtailment & efficiency behaviour**	*What do you do with the tap while tooth brushing*?	4	16
		*Which of the following water efficient appliances have you installed in your home*? *(water-saving shower head; high-efficiency washing machine; water saving device on kitchen tap)*	4	
		*What is the volume record on your latest water bill*?	8	
	**(IX) Tap water source protection**	*In the past 24 months*, *how did you dispose of your old medicines*?	6	10
		*In the past 24 months*, *how did you dispose of products such as old or used white spirit*, *stripper*, *brush softener or old weed killer*?	4	

(*) This question included the following explanation: *Anthropogenic substances end up in the environment through societal activities*, *and include substances originating from industry*, *agriculture*, *and households*, *such as pesticides*, *cleaning products*, *medicines and cosmetics*.

The framework constitutes of the dimensions cognition (head), attitude (heart), and behaviour (hands) and the substantive elements water quality, quantity and system. The nine combined components deriving from these dimensions and substantive elements are operationalised into survey questions.

## 4. Results

### 4.1 Tap water awareness assessment framework

As explained, this study’s conceptualisation of TWA distinguishes between cognitive awareness (head), affectional awareness (heart), and behavioural awareness (hands), and the elements water quality, quantity and system. Table 3 shows the resulting framework consisting of nine components, ranging from (I) ‘water quality comprehension’, relating to cognition and water quality, (V) ‘caring for water’, relating to affection and water quantity, to (IX) ‘tap water source protection’, relating to behaviour and water system. All nine components, as well as their operationalisation into research questions, are derived from an in-depth literature review as described below.

#### 4.1.1 Cognition (Head)

Cognitive TWA refers to knowledge, inquiry and understanding. Accordingly, it relates to the components (I) water quality comprehension, (II) water consumption knowledge, and (III) water system understanding. The leading assumption related to the cognitive dimension is that the more people know about tap water, be it in terms of quality, quantity, and/or system, the higher their awareness.

Our framework contains three questions to assess peoples’ ***water quality comprehension*** (component I). Building on the work of Gholson et al. [23] and Wang et al. [13], one question asks about the presence of anthropogenic substances in drinking water. Unlike, for instance the work of Gholson et al. [23] who in their study on consumer evaluations of public and private wells in Texas (United States), ask respondents which of a list of pollutants they knew (or suspected) to be threatening the water quality, it concerns a more general question based on the work of Brouwer et al. [38], with the answer categories of “none”, “a small quantity”, or a “large quantity” (see Table 3). Furthermore, the survey contains one cognitive question about the purification procedure and the use of chlorine. The latter question is especially relevant in the Netherlands where, in contrast to most other countries, tap water is distributed without disinfectant residuals [45]. One last question related to the water quality comprehension of customers is, among others, based on the work of Doria [27] and is about quality requirements of tap water versus bottled water.

***Water consumption knowledge*** (component II) is assessed by incorporating questions addressing both societal water use and the use of specific household appliances. Although the relation between water consumption knowledge, environmental issues, attitudes and behaviour is not always causal [46], Koop et al. [47] observe that having a solid knowledge base on individual and societal water consumption is generally considered to have a positive effect on conservation. At the same time, they identified an overall contradictory pattern of well-educated people stating to be more committed to water conservation compared to less formally schooled people, whilst consuming more water (i.e., the knowledge-behaviour gap). By assessing cognition, attitude and behaviour, our assessment framework helps to further elucidate this relationship. Building on the work of Willis et al. [24], our framework includes one question about the water use of a conventional shower head, and one question about the societal average daily water consumption.

In addition to assessing customers’ knowledge on water quality and water consumption issues, the framework also includes a number of questions about ***water system understanding*** (component III). Knowledge on the water supply system was also an integral aspect of the study of Petrescu [14], who studied environmentally-oriented behaviour in Romania. Building on this study, our framework assesses how well-informed people are about their drinking water utility’s name and responsibilities. Other aspects linked to water system understanding are incorporated in our framework through a knowledge question on the price and origin of tap water.

#### 4.1.2. Affection (Heart)

In the framework of Sipos et al. [32], heart refers to the enablement of the affective domain in forming values and attitudes, and also in our framework affective TWA relates to emotions, attitudes, interests, and feelings of belonging. Notwithstanding that tap water may not be well-known to elicit a variety of affective responses, we consider affection an integral element of TWA. Indeed, it is claimed that affection and emotions determine what we pay attention to, what we value, and, connected with the third element of our framework, how we behave [31]. Whereas the cognition (head) dimension relates to the question how much people know about tap water, the affective (heart) dimension is about how much people actually care about water. Accordingly, affective TWA relates to the components (IV) water quality perception, (V) caring for water consumption, and (VI) sense of responsibility. The leading associated assumption is that the less people take tap water for granted, the higher their TWA.

To assess peoples’ affection for tap water quality, our framework in the first place asks for the ***perceived quality of water*** (component IV), and accordingly determines the possible gap between actual and perceived water quality, i.e. to the extent to which the public believes that water is safe and of good quality [48]. Whereas experts approach risks with logic, reason, and scientific analysis, the general public relies more on heuristics, feelings, and quick, instinctive and intuitive responses to risk [38, 49, 50]. Accordingly, it is of no surprise that most people evaluate water quality differently than experts [51]. Nonetheless it is argued that it is of paramount importance not to disregard these public perceptions; ignoring them may result in public dissatisfaction or even undermine public confidence [22, 52]. Research suggests that the perceived quality and safety of water is dependent on a combination of several interrelated variables, including: (I) the organoleptic qualities of water, i.e. the characteristics of water that affect our senses of taste, smell and sight; (II) personal experiences, including personal memories of (health) problems; and (III) information from third parties, including media [22, 53]. In addition, (IV) research indicates that the level of trust in water utilities and regulatory authorities can have a significant impact on public trust in the quality and safety of drinking water [22, 38, 53–55]. Along with the perceived quality of water, our framework contains two questions related to the extent that people take access to clean drinking water for granted. Research suggests that access to clean drinking water is taken for granted in most high-income nations [56]. In the same vein, Hegger et al. [57] argue that tap water is considered a low-interest product. Building on this line of research, our framework assesses how frequently people ponder on the quality of their tap water, as well as to what extent they take the availability of clean tap water for granted.

Questions addressing the extent that people take drinking water for granted are also incorporated in ***caring for water*** (component V), in this case with respect to water quantity. More specifically, our framework includes a question to what extent people take it for granted that their tap provides safe potable water 24 hours a day. In addition, our framework assesses the degree to which people care and are aware about their water consumption. To this end, one question is included on how often people think about how much water they consume. Finally, caring for water is assessed by looking at their so-called intentional conservation behaviour. In the literature, knowledge and attitudes are frequently causally linked to behaviour. A leading theory in this respect is the Theory of Planned Behaviour (TPB), which postulates behaviour attitudes, subjective norms, and perceived behavioural control as major factors affecting behavioural intentions [58]. Given that this *behavioural intention* is regarded as a direct antecedent for the actual behaviour, from a TPB perspective, behavioural intention would be an important predictor of actual water saving behaviour.

In addition to assessing peoples’ perceived quality of water and caring for water, the framework also includes two tap water system questions under the denominator of ***sense of responsibility*** (component VI). This sensed responsibility may be conveyed into responsible action of consumers themselves to act in a water conserving or non-polluting way, as normative pressures are found to appeal pro-social behaviours [47, 59]. Our framework assesses whether people reflect on the origin of their water [23], and the degree to which they feel a personal responsibility to contribute to the protection of their drinking water sources. The underlying assumption related to sense of responsibility is that the more people feel associated and personally responsible, the higher their awareness is.

#### 4.1.3. Behaviour (Hands)

Besides determining the knowledge of and attitudes towards tap water quantity, quality and system issues, the framework also considers consumer behaviour as pivotal. Behavioural components of TWA relate to people’s action in practice, and pertain to behaviour and practices, consumption patterns as a part of daily life. Accordingly, it relates to the components (VII) quality-driven behaviour, (VIII) curtailment & efficiency behaviour, and (IX) tap water source protection. The explicit consideration of actual behaviour in our framework is in line with the most recent insights from the behavioural science literature. It is increasingly recognised that behavioural intention may not be the primary determinant of behaviour, but just one of the many factors [60, 61]. Accordingly, Kahneman [61] posits that two distinct cognitive systems (System 1 and System 2) are invoked during human decision-making: system 1, which is automatic, energy efficient, quick, and based on intuition, emotions and rules of thumb; and system 2, which is reflective, energy consuming, slow, intentional and based on ratio.

Our framework contains two questions to assess peoples’ ***quality-driven behaviour*** (component VII). One question relates to the active search for water quality information, with the underlying assumption that the more active people are in this respect, the smaller the gap between the actual risks and the risks they perceive. The lower this risk-perception gap, the higher their awareness. Building upon the work of Wang et al. [13], the second question asks about the consumption of bottled non-sparkling water at home. Bottled water is not only more expensive and less convenient, but more importantly, particularly troubling from an environmental perspective [62, 63]. Studies exploring the beliefs and motives that lead people to purchase bottled water suggest that a combination of factors, including lifestyle, trust in water utilities, and perceived alternatives, are all correlated with bottled water consumption, whereby organoleptics and concerns about health and water quality are particularly salient [27, 50, 62]. This study’s associated underlying assumption is clear: the higher one’s consumption of non-sparkling bottled water, the lower their TWA.

***Curtailment & efficiency behaviour*** (component VIII) is in the first place assessed by incorporating a question about a daily water-use pattern: tooth brushing. The question asks whether people close the tap during this activity. Previous study indicates that the annual wasted amount of water during tooth brushing may be relatively low in litres [64], but at the same time it is a common habit [40]. For instance, Ahmed [65] reports about a survey conducted in Britain showing that 72% of age group of 18–24 admits for water wastage while brushing teeth daily. In addition to this water-use pattern, and building on the work of Russell and Fielding [39], our framework refers to in-house water-saving appliances, and asks what types of appliances the respondent has installed at home, including a water-saving shower head and a high-efficiency washing machine. As to avoid a false distinction between tenants and buyers, we have opted for appliances that can be installed without major modifications or investments. Also, we have only included installations that do not apply for a specific type of housing. As such we have not included installations such as rainwater harvesting in gardens. Finally, the framework asks respondents to report the volume record on their latest water bill, as was also done by Willis et al. [24] and Fan et al. [66]. The associated underlying assumption is clear: a higher efficient and water-saving behaviour equals a higher TWA.

The last component of our TWA framework looks at behaviour from a water system perspective, and specifically focusses on ***tap water source protection*** (component IX), with the underlying assumption that the more people act protective and responsible, the more aware they are. To assess the extent to which people behave and act according to a consequence awareness in relation to water sources, the framework asks about disposal behaviour in relation to old medicines and products such brush softener or old weed killer, which are known to endanger the sources of drinking water [67]. The underlying reasoning resonates with some key elements of the Value-Belief-Norm model, which assumes that individual norms are key for adopting (altruistic) behaviour [68].

### 4.2 Tap water awareness in the Netherlands

As shown in Table 4, the results of this study show that the average Dutch citizen has a TWA score of 53.5%, meaning that, on average, people reached 53.5 points of the maximum total awareness points within the systematics of our framework. Especially people’s cognitive TWA, relating to one’s (I) water quality comprehension, (II) water consumption knowledge, and (III) water system understanding reveals, with an average score of 44.9%, ample room for improvement. For instance, about one third (30%) of all people lack basic familiarity with the name of their water utility, and a larger part (70.6%) does not know that the quality requirements for tap water are substantially more strict than for bottled water in the Netherlands. Slightly better are the scores for both affective (56.9%) and behavioural (60.3%) TWA, respectively relating to (IV) one’s water quality perception, (V) caring for water consumption, (VI) sense of responsibility, and people’s action in practice (components VII, VIII & IX). At this point it should be noted that relatively few respondents filled out the only non-compulsory question in our framework about their volume record on their latest water bill. In addition, a large share of the answers provided to this question were considered improbably high or low (above 400 or below 60 litres per household), so that from a reliability point of view they had to be disregarded. Given that after this correction only 33.0% of the respondents provided a reliable answer to the question of water use, this questions was disregarded in the analysis of Dutch water customers. Consequently, in the TWA assessment presented in this study, both the maximum number of points for the behavioural dimension and the water quantity component was lowered with 8 points. In consequence, the maximum score of awareness points was lowered from 108 to 100.

**Table 4:** Customer dimension awareness scores.

Dimension	Total score	Gender n = 1001	Age (n = 996)							Education (n = 1000)			Perspective (n = 999)			
			≤17	18–24	25–34	35–44	45–54	55–64	65≥	Low	Medium	High	Quality & health concerned	Aware & committed	Egalitarian & solidary	Down to earth & confident
**Total**	53.5	♀57.7*** ♂48.6 *t* = 8.76 (s)	53.1	52.5	51.7	52.6	55.6	53.9	52.0 *t* = -2.52 (vs)	52.0* t = -3.72 (s)	53.1	55.1*** t = 2.68 (vs)	52.2	56.9*** t = 6.85 (s)	53.7	50.0*** t = -5.71 (s)
**Cognition**	44.9	♀48.3 ♂41.0	41.0	46.3	42.7	43.0	45.5	45.1	47.9	40.8*** t = -5.72 (s)	44.1	48.4*** t = 4.69 (s)	41.0* t = -2.90 (vs)	46.9* t = 3.12 (s)	43.2	46.2
**Affection**	56.9	♀59.8* ♂53.6 *t* = 9.50 (s)	60.1	59.3	55.5	55.1	60.3	58.0	55.4	55.6	56.3	58.4* t = 2.50 (vs)	58.9	62.0*** t = 7.54 (s)	58.4	48.1*** t = -11.96 (m)
**Behaviour**	60.3	♀67.2 ♂52.0	59.6	55.5	58.4	61.7	62.5	60.1	62.5	61.7	60.3	59.3	58.0	63.0** t = 3.48 (s)	61.1	57.1

Significance:

* = *p* < .05;

** = *p* < .01;

*** = *p* < .001.

Depicted are percentages of maximum number of points.

As the differences between men and women in Table 4 shows, the latter have a substantial and significantly higher tap water consciousness (*p* < .001), a difference that primarily relates to the affectional dimension (*p* < .05). As expected, significant differences were also found in the educational background of respondents. Respondents with a low education show a significantly lower overall TWA (*p* < .05). This lower awareness score, however, can solely be explained by a significant lower cognitive tap water consciousness (*p* < .001), and does not relate to the affective nor to the behaviour dimension. By contrast, citizens with a high education show a significantly higher overall TWA. This difference relates most strongly to a significantly higher cognitive TWA (*p* < .001) and to a lesser extent to a significantly higher affectional TWA. Strikingly, the differences between the different age groups are negligible. Additional TWA scores for demographic variables are presented in S3 Appendix.

From a modern segmentation perspective, and an analysis on the possible differences on the basis of the four earlier introduced customer perspectives on drinking water as presented in Table 1, we find that the respondents with the ‘aware & committed’ perspective show a significantly higher overall TWA (*p* < .001). On the contrary, respondents with the ‘down to earth & confident’ perspective show a significantly lower overall tap water consciousness (*p* < .001). Indeed, and in support of the work of Brouwer et al. [25], this study shows that respondents with the ‘aware & committed’ perspective highly value sustainable behaviour, including, for instance, water-saving efforts, whereas respondents with the ‘down to earth & confident’ perspective are characterized by great confidence in the responsibility of drinking water utilities, along with the desire not to be bothered about drinking water. When zooming in further on the results depicted in Table 4, it can be observed that not only the total awareness scores reach this significance, but also all three separate awareness dimensions. Indeed, respondents with the ‘aware & committed’ perspective show a significantly higher cognitive (*p* < .05), affective (*p* < .001), and behavioural awareness (*p* < .01). By contrast, respondents with the ‘down to earth & confident’ perspective only show a highly significantly lower affectional awareness (*t* = -11.961, *p* < .001). Interestingly, respondents with the ‘down to earth & confident’ perspective do not show a lower cognitive TWA. On the contrary, although respondents with the ‘aware & committed’ perspective show the highest score, respondents with the ‘down to earth & confident’ perspective show a slightly higher cognitive tap water consciousness. This is a telling result, for it suggests that respondents with the ‘down to earth & confident’ perspective show a relative low behavioural TWA. Not because they have less water knowledge, inquiry or understanding than respondents with the other perspectives, but simply because they care less. A partly opposite result can be seen in respondents with the ’quality & health concerned’ perspective, characterised by their focus on personal preferences and needs, especially regarding their own health. These customers show a lower cognitive tap water consciousness. However, at the same time they show a higher affectional tap water consciousness.

Table 5 depicts the scores of Dutch drinking water customers for the nine different awareness components of our three-by-three dimensioned TWA assessment framework, organised around the principles head (cognition), heart (affection), and hands (behaviour), and the substantive themes water quality, water quantity and system. As explained, the scores along the layout of the dimensions shows that the average scores are highest for behavioural and affective TWA, and relatively low for cognitive TWA. As shown in S3 Appendix, from a thematic point of view, we find that people’s water quality awareness is, with an average score of 48.2%, relatively low, followed by water quantity with an average score of 54.7%. People’s water system awareness, relating to awareness of tap water in its broader context, scores relatively best with an average score of 57.9%.

**Table 5:** Customer component awareness scores—Gender and age.

Dimension	Component	Total score	Gender (n = 1000)	Age (n = 996)						
				≤17	18–24	25–34	35–44	45–54	55–64	65<
**Cognition**	**(I) Water quality comprehension**	40.7	♀42.9 ♂38.2 *t* = 2.57	32.0	41.9	38.7	42.2	40.0	38.8	44.6
	**(II) Water consumption knowledge**	34.2	♀35.7 ♂32.4 *t* = 2.58	30.0	33.0	30.9	32.4	37.1	35.9	36.9
	**(III) Water system understanding**	52.0	♀56.8*** ♂46.3 *t* = 7.76 (s)	50.9	54.7	50.1	48.2	52.2	52.7	54.6
**Affection**	**(IV) Water quality perception**	55.7	♀56.7* ♂54.5 *t* = 2.66 (vs)	57.5	56.5	54.4	54.5	57.0	55.6	55.8
	**(V) Caring for water**	57.8	♀61.9*** ♂52.9 *t* = 6.78 (s)	62.5	55.9	55.6	55.3	63.1	60.9	53.9
	**(VI) Sense of responsibility**	58.2	♀62.8 ♂52.7	61.7	56.7	57.4	56.2	62.9	58.3	56.6* t = -2.44 (vs)
**Behaviour**	**(VII) Quality-driven behaviour**	43.8	♀44.4 ♂43.2	46.0	44.3	42.6	42.6	46.2	43.3	44.3
	**(VIII) Curtailment & efficiency behaviour**	70.7	♀74.3*** ♂66.5 *t* = 6.00 (s)	72.1	66.7	71.2	69.4	71.1	70.9	73.4
	**(IX) Tap water source protection**	68.3	♀84.5*** ♂49.2 *t* = 20.16 (l)	63.3	57.7* t = -2.87 (vs)	64.1	74.6	72.0	68.3	72.1

Significance:

* = *p* < .05;

** = *p* < .01;

*** = *p* < .001.

Depicted are percentages of maximum number of points.

Looking at the individual awareness components as detailed in Tables 5 and 6, we find a rather wide variation in maximum awareness scores, ranging from 34.2% for component (II) water consumption knowledge to 70.7% for component (VIII) curtailment & efficiency behaviour. Indeed, based on the survey answers on the questions about the water use of a conventional shower head and the societal average daily water consumption, it appears that customers generally have “cognitive gaps” in their understanding of water consumption knowledge. For one illustration, on the open question of estimating the average daily water consumption of one person in the Netherlands, only 5% was able to give the correct answer of 120 litres (with a margin of ten litres). Most citizens, however, estimate this consumption below 50 litres (51%), between 91–110 litres (10%), or between 131–150 litres per day (6%). On the other end, we also find a group of respondents (16%) estimating that this average daily consumption of water is more than 191 litres a day. We found that respondents with a high level of education have a higher tap water consumption knowledge. Water consumption knowledge is the only water awareness component without a significant correlation with one of the four water customer perspectives.

**Table 6:** Customer component awareness scores—Education and perspective.

Dimension	Component	Total score	Education (n = 1000)			Perspective (n = 999)			
			Low	Medium	High	Quality & health concerned	Aware & committed	Egalitarian & solidary	Down to earth & confident
**Cognition**	**(I) Water quality comprehension**	40.7	37.9	38.5	44.9** t = 3.37 (s)	35.8	45.5** t = 3.80 (s)	38.9	39.2
	**(II) Water consumption knowledge**	34.2	29.9** t = -3.38 (s)	34.5	36.7* t = 2.92 (vs)	32.7	35.2	33.3	34.8
	**(III) Water system understanding**	52.0	47.2*** t = -4.10 (s)	51.5	55.6*** t = 4.51 (s)	47.6* t = -2.63 (vs)	52.9	49.9	55.3** t = 3.41 (s)
**Affection**	**(IV) Water quality perception**	55.7	52.4*** t = -4.62 (s)	55.7	57.7*** t = 4.43 (s)	53.3	58.9*** t = 5.74 (s)	55.8	52.6*** t = -3.76 (s)
	**(V) Caring for water**	57.8	58.5	57.4	57.6	61.4* t = 2.50 (vs)	63.6*** t = 5.62 (s)	61.8*** t = 3.67 (s)	44.5*** t = -12.11 (m)
	**(VI) Sense of responsibility**	58.2	57.8	56.0	60.8	66.1*** t = 4.19 (s)	65.9*** t = 5.68 (s)	58.5	44.5*** t = -10.58 (m)
**Behaviour**	**(VII) Quality-driven behaviour**	43.8	39.4** t = -3.33 (s)	43.1	47.6*** t = 4.01 (s)	41.7	48.0** t = 3.71 (s)	41.7	42.0
	**(VIII) Curtailment & efficiency behaviour**	70.7	69.9	71.8	70.1	70.6	72.8 ^1^	72.1* t = 2.05 (vs)	66.8
	**(IX) Tap water source protection**	68.3	77.5*** t = -5.03 (s)	68.4	62.4*** t = 4.77 (s)	64.1	70.0	71.9* t = 2.25 (vs)	64.5

Significance:

* = *p* < .05;

** = *p* < .01;

*** = *p* < .001.

Depicted are percentages of maximum number of points.

^1^ The average curtailment and efficiency behaviour score of respondents with ‘aware & committed’ perspective is higher, though not significant, as compared to respondents with the ‘egalitarian & solidary’ perspective. This can be explained by the difference in STD. With an average of 5.656, the former segment has a STD of 0.35521, whereas the latter segment has a STD of 0.35047.

The component with the highest awareness scores relates to (VIII) **curtailment & efficiency behaviour**, assessed by asking people about their daily tooth brushing water-use pattern, their water-saving appliances installed in-house and in theory, but as explained above disregarded, the volume record on their latest water bill. The high score can mainly be explained by the tap water use while tooth brushing. 76.4% of the respondents state to always close the tap while brushing their teeth and another 15.8% almost always does so. Moreover, people also stated to have (several) water saving appliances installed in their homes. 54.6% has a water saving showerhead and 59.4% has a water saving washing machine. Women show significant and substantial higher levels of curtailment & efficiency behaviour than men (*p* < .001).

A third awareness component worth singling out is component (IV) **water quality perception**. Not because this component has resulted necessary in deviant scores but because, next to quality perception and the question on the frequency of thinking about the quality of water, it assessed to what extent respondents perceive clean tap water as something that can be taken for granted. A small majority of 50.6% of the respondents ’fully agrees’ and a further 37.4% ’agrees’ with the statement that clean tap water is something obvious. The percentage of respondents that instead do not regard clean tap water as something obvious, but instead for instance as rather special, is very small. Only 4.9% does not agree with the former statement; 0.8% does not agree at all. Overall, we find that again women score higher on water quality perception than men do. Also respondents with a high education have an overall slightly higher water quality perception. Again the differences are bigger when looking at the different customer perspectives. Respondents with the ‘aware & committed’ perspective show a significantly higher water quality perception (*p* < .001). On the other end, customers classified according to the ‘down to earth & confident’ (*p* < .001) and to a lesser extent also the ‘quality & health concerned’ perspective, have a significantly lower water quality perception. The reason why these two groups score low on this aspect, however, differs per perspective. Respondents with the ‘down to earth & confident’ perspective score especially low because they overwhelmingly consider it self-evident that clean water runs out of the tap. Respondents with the ‘quality & health concerned’ perspective, on the other hand, score particularly low because they—unjustly—relatively often have the idea that the quality of their tap is uncertain.

**Fig 1:**
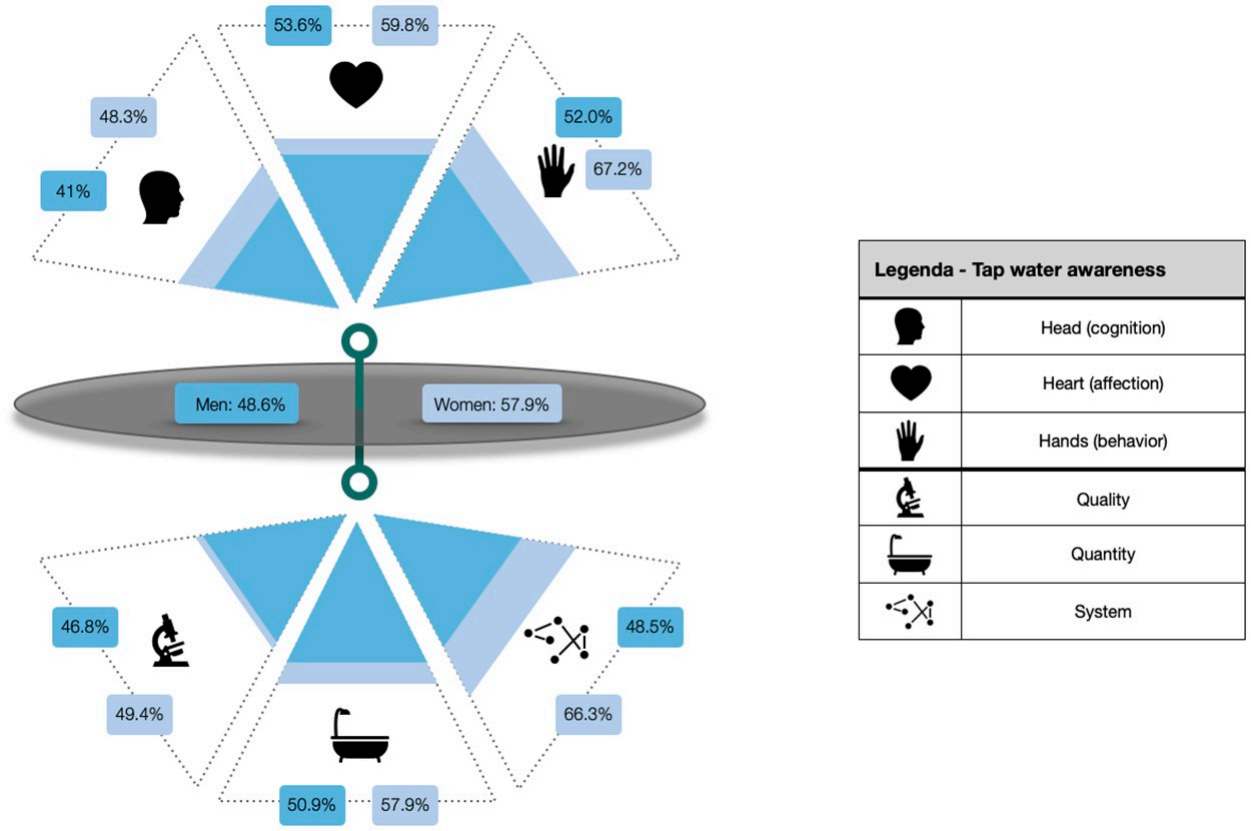
Illustration of the aggregated tap water awareness profile for both women and men.

Overall it is remarkable that women score higher than men on all components, albeit not always significantly. The individual scores for the head (cognitive), heart (affective), and hands (behavioural) dimension are presented in Tables 5 and 6. Fig 1 illustrates these different scores into a TWA profile. The strongest difference between women and men relates to the tap water source protection (IX) scores (84.5% versus 49.2%, *t* = 20.161, *p* < .001), in our framework assessed by asking respondents about their medicine and chemical products disposal behaviour.

Next to the gender difference, the biggest differences relate to the different perspectives. Indeed, respondents with the ‘aware & committed’ perspective have a significantly higher TWA (*p* < .001). This finding is reflected in Table 6, depicting that this segment shows a higher water quality comprehension (I) and water quality perception (IV). Furthermore, the results of this study show that respondents with the ‘aware & committed’ perspective care significantly more for water, and depict a higher caring for water (V) and sense of responsibility (VI) score, and have significantly higher levels of quality-driven behaviour (VII). The segment with relative the lowest TWA are respondents with the ‘down to earth & confident’ perspective. Table 6 shows that this particularly relates to a lower affectional scores. Indeed, we find that these customers not only have a significantly lower water quality perception (VI), but also care significantly less for water (v), and feel a significantly lower sense of responsibility (for all three, *p* < .001). As indicated in the above, this result is not related to a lower cognitive score, if only because this very segments shows a significantly higher water system understanding (III; *p* < .001).

As already noted, a partly opposite result can be seen in respondents with the ’quality & health concerned’ perspective. These customers show a lower water system understanding (III). At the same time, they tend to care more for water (V), and alike have a higher sense of responsibility (VI). The solely affectional component where respondents with the ’quality & health concerned’ perspective show a lower score is tap water quality perception (IV). This finding can, however, be fully explained by the elemental position of the profile itself, in which care for and concern about the quality of water in relation to their health are central [25]. Finally, we find that respondents with the ‘egalitarian & solidary’ perspective have a significant higher score on two components of the behavioural dimension, i.e. curtailment & efficiency behaviour (VIII) and tap water source protection (IX). In addition, they also show significantly more care for water, but this relates more to the very low score of respondents with the ‘down to earth & confident’ perspective than to a remarkably high score for ‘egalitarian & solidary’ customers.

Looking at the differences between respondents with different educational backgrounds, depicted in Table 6, especially the behavioural component is noteworthy. Here we find that respondents with a low level education have significantly lower levels of quality-driven behaviour (*p* < .01) but substantially higher levels of tap water source protection (IX; *p* < .001). Interestingly, we find the reversed patterns when looking at respondents with a high education. Indeed, they show significantly higher levels of quality-driven behaviour (VII; *p* < .001) and significantly lower levels of tap water source protection (IX; *p* < .001). This contradiction is also observed in other studies [47, 66].

## 5. Discussion and conclusion

This study shows that a complex and multifaceted concept such as TWA, which seems to have become a catch-all term, can be well conceptualised and operationalised into a practicable empirically-based assessment framework. Consistent with the organizing principle of head, heart, and hands, this three-by-three dimensioned assessment framework distinguishes between cognitive, affective and behavioural awareness of tap water. In addition to this threefold conceptualisation, a second distinction was made based on the substantive characteristics of tap water: water quality, water quantity and water system.

### 5.1 Key observations

In addressing the research aim to conceptualise, operationalise and assess TWA, this study has presented a TWA framework consisting of three dimensions; nine components; and 24 questions, which subsequently has been empirically tested in a large-scale survey in the Netherlands. Overall, this assessment has demonstrated that, as previously observed by the OECD [12], TWA in the Netherlands shows ample room for improvement. This is especially the case for the cognitive dimension of TWA, relating to one’s water quality comprehension, consumption knowledge, and system understanding. The scores for both affective and behavioural tap water awareness are slightly higher, with the components ’tap water source protection’ and ’curtailment & efficiency behaviour’ as positive outliers.

The tap water assessment analysis becomes even more interesting when we break down the scores for the different dimensions and components to different types of customers. It is striking that women score higher than men on most aspects of TWA, whereas other socio-demographic variables were not or hardly distinctive. The former observation is in coherence with previous studies demonstrating that women consume less tap water as compared to men [69, 70] as well as with studies suggesting that, on average, women in the Netherlands make more sustainable behavioural choices [71]. Segmentation based on the drinking water customer perspectives show, as previously reported by, for instance, Brouwer et al. [38] and Koop et al. [72], clear differences all along the line. Customers with the ‘aware & committed’ perspective have significantly higher, and customers holding a ‘down to earth & confident’ perspective significantly lower TWA scores.

The combined insight into the different customer perspectives and the proposed assessment framework may facilitate both the effective implementation and evaluation of future TWA raising campaigns. After all, for policymakers it is important to acknowledge that TWA consists of more than caring for water alone, and that e.g. the tap water knowledge people may have not always aligns with their conservation efforts or quality protecting behaviour. Moreover, it is important to appreciate that techniques that may work well for customers with, for instance, the ’aware & committed’ perspective may generate an entirely different effect on customers holding a ‘quality & health concerned’ perspective.

### 5.2 Underlying *tap water awareness* patterns in the Netherlands

**Fig 2:**
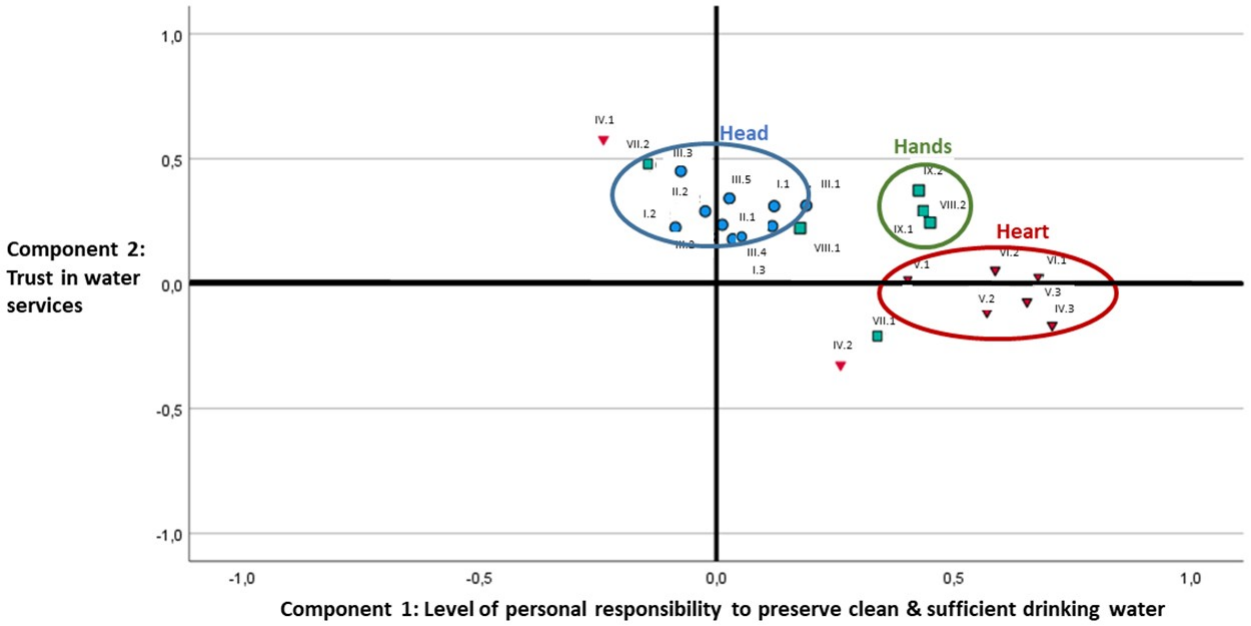
Component plot of the two dominant components that together explain 21.2% of the variance.

Beyond this study’s primary objective, the rich survey results provide ample opportunity to explore underlying patterns and key factors that characterise Dutch TWA. As such, both a Principle Component Analysis (PCA) and a multiple regression analysis have been conducted. The PCA has identified two main components explaining 21.2% of the variance (see S4 Appendix). Component 1 represents a pattern where respondents think relatively often about the quality (question IV.3), origin (question VI.1) and own consumption of drinking water (question V.3), experience tap water services as special (question V.2), have water-saving household devices (question VIII.2), want to save water (question V.1) and dispose leftover medicines, paint and pesticides in an environmentally friendly manner (question IX.1 and IX.2). This coherence in the affection and behaviour related to the substantive elements water quantity and system suggest ‘a level of personal responsibility to preserve clean and sufficient drinking water’. Interestingly, the perception and behaviour of water quantity and systemic responsibilities seem therefore intertwined irrespective of people’s knowledge about the topic. Component 2 consisting of knowledgeability of the responsibilities of drinking water utilities (high score question III.3), perception that drinking water is safe (high score question IV.1), and low consumption of bottled water (high score question IV.2) may represent ‘a trust in water services’. Plotting these two key components shows a clear distinction between head, heart and hands dimensions (Fig 2).

Overall we can observe that the level of personal responsibility for preserving clean and sufficient drinking water is positively related to heart and to a lesser extent with hands. Interestingly, the level of knowledge is only positively related with the trust in water services. The more people know about drinking water, the higher their trust in these services. However, knowledge has no positive or negative impact on the level of personal responsibility people experience to preserve clean and sufficient drinking water. From this observation, it may be reasoned that campaigns that only focus on enhancing people’s knowledge may improve people’s trust in water services, but generate little or no impact on the level of personal responsibility to help preserve this service by for instance reducing their water use or by more environmentally-considered disposal of their leftover medicines, paint or pesticides.

A multiple regression analysis was applied with the aim of exploring which questions are most predictive for the overall score. As a result, the following questions turned out to be the most predictive for the overall TWA score: questions on (i) the disposal of leftover medicines (question IX.1); (ii) the consumption of bottled water (question VII.2); (iii) the knowledge that there is no chlorinate in the Dutch drinking water (question I.2); (iv) the knowledge that quality requirements are more stringent for tap water than for bottled water (question I.3), and (v) the knowledge on the origin of tap water (question III.1). A key observation of these most predictive questions is that the quality requirements and measures taken to produce drinking water are relatively unknown, also in relation to bottled water (low scores on questions I.3, I.2), whilst the related behaviour of drinking bottled water at home still occurs regularly (question VII.2). This misconception on bottled water seems to be particularly telling in understanding someone’s tap water awareness. Further details of the multiple regression analysis are provided in S4 Appendix.

### 5.3 Avenues for future research

Future research is needed to determine which strategies can best be used to increase the TWA of different types of customers. These strategies may combine several TWA dimensions. For example, the roll-out of digital water meters in combination with frequent user feedback in terms of both litres and costs may appeal to both the cognitive and the behavioural dimension [72, 73]. For both the affective and cognitive TWA dimension, the implementation of citizen science projects in the field of tap water could cause customers to experience that the self-evidence of constant clean tap water may, in fact, be rather special [74]. Finally, the use of smart behavioural techniques such as the use of emotional shortcuts and nudging [47] may form promising strategies, appealing both the affective and behavioural dimension of awareness. In addition, it is interesting to work towards a comparable—and eventually integrated assessment framework—for also surface and groundwater. Additional challenges that need to be addressed in the future relate to the fact that the current study and the framework is not without limitations. First, while the gap between intentional and actual behaviour is acknowledged, the present study builds on self-reported behaviour. Second, relatively many respondents did not report their actual water consumption by checking the volume record on their latest water bill, impeding an analysis of actual consumption patterns in relation behavioural intentions, i.e. the so-called “intention-behaviour gap” [24, 75]. Third, the context in which this research was conducted—that is, a small and wealthy country marked by its high quality of tap water and publicly owned utilities—might have influenced our results. After all, Koop et al. [76] suggest that local contextual factors, such as the different risks, probabilities and impacts, past experiences, existing institutions and policies may considerably impact people’s water awareness. Future studies could constructively build on the current research by (a) assessing actual behaviour, (b) with consent of respondents, coupling survey results to actual user data provided by utilities, and (c) applying the assessment framework in other contexts and cultures.

## Supporting information

S1 Appendix TWA questions and weighing system. (DOCX)

S2 Appendix Results per question. (DOCX)

S3 Appendix Additional TWA scores. (DOCX)

S4 Appendix PCA and multiple regression. (DOCX)
