# Supplementary material for: Assessing tap water awareness: The development of an empirically-based framework
Source: PLoS One. 2021 Oct 29;16(10):e0259233. doi: 10.1371/journal.pone.0259233 (PMC8555835; doi:10.1371/journal.pone.0259233)
Supplement: S1 Appendix — (DOCX) [file pone.0259233.s001.docx]

**Appendix – TWA questions and weighing system**

| **Component** | **No.** | **Operational questions** | **Weighing** | |
| --- | --- | --- | --- | --- |
| **(I) Water quality comprehension - Cognition** | *I.1* | *My tap water contains… of anthropogenic substances** | - *None (0)* - *A small quantity (2)* - *A large quantity (0)* - *Don’t know (0)* | 2 |
|  | *I.2* | *To your knowledge, is chlorine added to your tap water?* | - *Yes (0)* - *No (4)* - *Don’t know (0)* | 4 |
|  | *I.3* | *Are the quality requirements stricter for tap water or bottled water?* | - *Bottled water (0)* - *Tap water (4)* - *The quality requirements are similar (0)* - *Don’t know (0)* | 4 |
| **(II) Water consumption knowledge - Cognition** | *II.1* | *Estimate the average daily water consumption of one person in the Netherlands?(open question)* | *110-130 liters (4); 90-110 or 130-150 liters (3); 70-90 or 150-170 liters (2); 50-70 or 170-190 liters (1); <50 or >190 liters (0)* | 4 |
|  | *II.2* | *Estimate how much water, in litres, a conventional shower head uses per minute? (open question)* | *6-8 liters (4); 4-5 or 9-12 liters (3); 3 or 13-17 liters (2); 18-24 liters (1); <3 or >25 liters (0)* | 4 |
| **(III) Water system understanding - Cognition** | *III.1* | *What is the source of your tap water? (surface water, ground water, dune water; seawater; other, namely …; don’t know)* | *Check with postal code. Correct (2); wrong or don’t know (0)* | 2 |
|  | *III.2* | *What is the name of your drinking water utility?* | - *Check with postal code. Correct (including wrongly spelled)(4), wrong answer (0)* | 4 |
|  | *III.3* | *Which responsibilities do you think belong to the tasks of your drinking water utility (swimming water management; sewage treatment; tap water purification; provision of tap water; prohibiting dike breaches; management of sewage infrastructure)* | *Check with postal code (responsibilities differ per utility). Answer should always include ‘provision of tap water’; and ‘tap water purification’(if not 0 points are awarded). One point less per wrong answer.* | 4 |
|  | *III.4* | *Drinking water utilities are responsible for the quality of tap water up to the… pumping station; the water meter; the tap)* | - *Pumping station (0)* - *Water meter (4)* - *Tap (0)*   *Don’t know (0)* | 4 |
|  | *III.5* | *What is the price for 1,000 litres (1 m3) of tap water, excluding taxes?(open question)* | *€ 0,50-2,00 (4); € 0,25-0,50 or € 2,00-5,00 (3); € 0,10-0,25 or € 5,00-10,00 (2); € 0,05-0,10 or € 10,00 -20,00 (1); € <0,05 or €>20,00 (0)* | 4 |
| **(IV) Water quality perception - Affection** | *IV.1* | *How safe do you perceive tap water in the Netherlands?* | - *Very safe (8)* - *Safe (6)* - *Not safe, yet not unsafe (2)* - *Unsafe (1)* - *Very unsafe (0)* | 8 |
|  | *IV.2* | *In my view, clean tap water is something obvious* | - *Fully agree (0)* - *Agree (1)* - *Neutral (2)* - *Disagree (3)* - *Fully disagree (4)* | 4 |
|  | *IV.3* | *How often do you think about the quality of your tap water?* | - *With every use (4)* - *Daily (3)* - *Weekly (2)* - *Monthly (1)* - *Never (0)* | 4 |
| **(V) Caring for water - Affection** | *V.1* | *I would like to save (more) tap water at home* | - *Fully agree (4)* - *Agree (3)* - *Neutral (2)* - *Disagree (1)* - *Fully disagree (0)* | 4 |
|  | *V.2* | *Every single day I experience 24 hours running tap water as special* | - *Yes (4)* - *No (0)* - *Now I think about it: Yes (2)* | 4 |
|  | *V.3* | *How often do you think about your water consumption?* | - *With every use (4)* - *Daily (3)* - *Weekly (2)* - *Monthly (1)* - *Never (0)* | 4 |
| **(VI) Sense of responsibility - Affection** | *VI.1* | *I sometimes think about the origin of my tap water* | - *Fully agree (4)* - *Agree (3)* - *Neutral (2)* - *Disagree (1)* - *Fully disagree (0)* | 4 |
|  | *VI.2* | *I feel a personal responsibility for protecting the quality of water in rivers, lakes, ditches and subsurface* | - *No (0)* - *Yes (4)* - *Somewhat (2)* | 4 |
| **(VII) Quality -driven behaviour - Behaviour** | *VII.1* | *In the past 24 months, have you ever actively looked for information on the quality and safety of Dutch tap water (e.g. via the Internet or by contacting your drinking water utility)?* | - *Yes (4)* - *No (0)* | 4 |
|  | *VII.2* | *How often do you drink bottled non-sparkling water at home?* | - *Daily (0)* - *Multiple times per week (1)* - *Multiple times per month (2)* - *A number of times per year (4)* - *Never (6)* | 6 |
| **(VIII) Curtailment & efficiency behaviour- Behaviour** | *VIII.1* | *What do you do with the tap while tooth brushing?* | - *I always close the tap (4)* - *I almost always close the tap (3)* - *I sometimes close the tap (1)* - *I never close the tap (0)* | 4 |
|  | *VIII.2* | *Which of the following water efficient appliances have you installed in your home? (water-saving shower head; high-efficiency washing machine; water saving device on kitchen tap; other, namely …; none)* | *Number of appliances installed: 3 or more (4); 2 (3); 1 (2); none (0). The points for ‘other, namely…’ category were given for appliances that are applicable for all households (not only with a garden for instance).* | 4 |
|  | *VIII.3* | *What is the volume record on your latest water bill?* | *Based on the dates* an average daily use per person is calculated. >220 liter a day (0); 150-220 liter (2); 90-120 liter (4); 75-90 liter (2); 60-75 liter (8)***  ** records spanning under 3 months are excluded.*  ***Below 60 liters a day is dismissed as untrustworthy.* | 8 |
| **(IX) Tap water source protection - Behaviour** | *IX.1* | *In the past 24 months, how did you dispose of your old medicines?* | - *Leave at home / nothing (2)* - *Returned at the pharmacy, store or recycling center (6)* - *Disposed of in the trash can (1)* - *Flushed down the sink or toilet (0)* | 6 |
|  | *IX.2* | *In the past 24 months, how did you dispose of products such as old or used white spirit, stripper, brush softener or old weed killer?* | - *Leave at home / nothing (2)* - *Returned to the store, recycling center or collection point for small chemical waste (4)* - *Disposed of in the trash can (1)* - *Flushed down the sink or toilet (0)* | 4 |

**TWA in the original language (Dutch)**

| **Element** | **Survey vragen** | | **Antwoorden** |
| --- | --- | --- | --- |
| **(I) Begrip waterkwaliteit** | *I.1* | *Niet-natuurlijke stoffen zijn stoffen die door de samenleving in het milieu terecht komen. Bijvoorbeeld stoffen vanuit de industrie, de landbouw/veeteelt, ziekenhuizen en huishoudens zoals bestrijdingsmiddelen, schoonmaakmiddelen, medicijnen en cosmetica.*  In kraanwater zitten … | - *geen niet-natuurlijke stoffen* - *een kleine hoeveelheid niet-natuurlijke stoffen* - *een grote hoeveelheid niet-natuurlijke stoffen* - *weet ik niet* |
|  | *I.2* | Denkt u dat er aan uw kraanwater chloor wordt toegevoegd? | - *ja* - *nee* - *weet niet* |
|  | *I.3* | Denkt u dat in Nederland/Vlaanderen de kwaliteitseisen hoger liggen voor kraanwater of voor flessenwater? | - *Flessenwater* - *Kraanwater* - *De kwaliteitseisen liggen gelijk* - *Weet ik niet* |
| **(II) Kennis waterkwantiteit** | *II.1* | Spontaan, zonder op te zoeken. Schat in hoeveel kraanwater een persoon in Nederland/Vlaanderen gemiddeld per dag gebruikt | *… liter per dag* |
|  | *II.2* | Spontaan, zonder op te zoeken. Schat in hoeveel kraanwater er wordt gebruikt met 1 minuut douchen (standaard douchekop, geen bespaar, comfort of regendouche). | *… liter per minuut* |
| **(III) Begrip watersysteem** | *III.1* | Uit welke bron denkt u dat uw kraanwater wordt gemaakt? | - *Grondwater* - *Oppervlaktewater (bijv. uit een rivier of meer)* - *Duinwater* - *Zeewater* - *Anders, namelijk…* - *Weet niet* |
|  | *III.2* | Wat is volgens u de naam van uw drinkwaterbedrijf? | *Open vraag, met ‘weet niet’ optie* |
|  | *III.3* | Welke onderstaande verantwoordelijkheden behoren volgens u tot de taken van uw drinkwaterbedrijf? *Meerdere antwoorden mogelijk.* | - *Beheer van zwemwater* - *Zuivering van rioolwater* - *Schoonhouden grond- en oppervlaktewater* - *Voorkomen van dijkdoorbraken* - *Zuivering van kraanwater* - *Levering van kraanwater* - *Aanleg en beheer riolering* |
|  | *III.4* | Wat is volgens u het juiste antwoord?  Drinkwaterbedrijven zijn verantwoordelijk voor de kwaliteit van het kraanwater… | - *tot het pompstation* - *tot en met de voordeur (watermeter)* - *tot het uit de kraan komt* - *weet niet* |
|  | *III.5* | Wat denkt u dat de prijs is voor 1.000 liter (1 m3) kraanwater, exclusief belastingen? *Maak een zo goed mogelijke schatting.* | *…,… euro per 1000 liter* |
| **(IV) Perceptie van de waterkwaliteit** | *IV.1* | Hoe veilig ervaart u het kraanwater in Nederland/ Vlaanderen? | - *Zeer veilig* - *Veilig* - *Niet veilig, maar ook niet onveilig* - *Onveilig* - *Zeer onveilig* |
|  | *IV.2* | Voor mij is het vanzelfsprekend dat er schoon drinkwater uit de kraan komt. | - *Helemaal mee eens* - *Mee eens* - *Neutraal* - *Niet mee eens* - *Helemaal niet mee eens* |
|  | *IV.3* | Hoe vaak staat u stil bij de kwaliteit van uw kraanwater? | - *Dagelijks* - *Wekelijks* - *Maandelijks* - *Een aantal keer per jaar* - *(Bijna) nooit* |
| **(V) Geven om water** | *V.1* | Ik zou thuis graag (nog) meer kraanwater willen besparen. | - *Helemaal mee eens* - *Mee eens* - *Neutraal* - *Niet mee eens* - *Helemaal niet mee eens* |
|  | *V.2* | 24 uur per dag water uit de kraan ervaar ik iedere dag weer als bijzonder | - *Ja* - *Nee* - *Wel nu ik er over nadenk* |
|  | *V.3* | Hoe vaak denkt u er over na hoeveel water u gebruikt? | - *Bij ieder gebruik* - *Dagelijks* - *Wekelijks* - *Maandelijks* - *Bijna nooit* |
| **(VI) Verantwoordelijk-heidsgevoel** | *VI.1* | Ik denk wel eens na over de herkomst van mijn kraanwater | - *Helemaal mee eens* - *Mee eens* - *Neutraal* - *Niet mee eens* - *Helemaal niet mee eens* |
|  | *VI.2* | Ik voel een persoonlijke verantwoordelijkheid voor de bescherming van de waterkwaliteit van de ondergrond, sloten, rivieren en meren | - *Ja* - *Nee* - *Enigszins* |
| **(VII) Kwaliteits-gestuurd gedrag** | *VII.1* | Bent u in de afgelopen 24 maanden wel eens actief op zoek gegaan naar informatie over de kwaliteit en veiligheid van Nederlands/ Vlaams kraanwater (bijv. via internet of door contact op te nemen met het drinkwaterbedrijf)? | - *Ja* - *Nee* |
|  | *VII.2* | Hoe vaak drinkt u thuis flessenwater zonder prik? | - *Dagelijks* - *Meermaals per week* - *Meermaals per maand* - *Een aantal keer per jaar* - *Nooit* |
| **(VIII) Efficiëntie gedreven waterconsumptie** | *VIII.1* | Wat doet u met de kraan tijdens het tandenpoetsen? | - *Ik draai de kraan altijd dicht* - *Ik draai de kraan bijna altijd dicht* - *Ik draai de kraan soms dicht* - *Ik draai de kraan nooit dicht* |
|  | *VIII.2* | Welke van de onderstaande opties om kraanwater te besparen heeft u in uw woonhuis? (meerdere antwoorden mogelijk) | - *Besparende douchekop* - *Waterbespaarder op de keukenkraan* - *Zuinige wasmachine* - *Anders, namelijk…* - *Weet niet* - *Geen* |
|  | *VIII.3* | Bekijk uw laatste periodeafrekening van uw drinkwaterbedrijf. Hoeveel kraanwater heeft u de afgelopen periode gebruikt? Noteer de hoeveelheid water, en de 2 data waartussen deze hoeveelheid water is gebruikt. | *Hoeveelheid water: … m3 water*  *Startdatum periodeafrekening: … (dd/mm/yyyy)*  *Einddatum periodeafrekening … (dd/mm/yyyy)* |
| **(IX) Bescherming van kraanwater-bronnen** | *IX.1* | Wat heeft u de afgelopen 24 maanden gedaan met uw oude medicijnen? *(als u de afgelopen 24 maanden geen oude medicijnen in huis heeft gehad, geef dan antwoord op de vraag: “Wat zou u doen met oude medicijnen?) Meerdere antwoorden mogelijk* | - *Thuis laten liggen/niks* - *Ingeleverd bij de apotheek, winkel of milieustraat [Voor Vlaanderen: Ingeleverd bij de winkel, recyclagepark of inzamelpunt klein gevaarlijk afval (kga)]* - *Weggegooid in de vuilnisbak* - *Doorgespoeld door de gootsteen of het toilet.* |
|  | *IX.2* | Wat heeft u de afgelopen 24 maanden gedaan met producten zoals oude of gebruikte terpentine, afbijtmiddel, kwastontharder of oude onkruidverdelger? *(als u de afgelopen 24 maanden niet dit soort producten in huis heeft gehad, geef dan antwoord op de vraag: “Wat zou u doen met producten zoals oude of gebruikte terpentine, afbijtmiddel, kwastontharder of oude onkruidverdelger?) Meerdere antwoorden mogelijk* | - *Thuis laten liggen/niks* - *Weggooien in de vuilnisbak* - *Doorspoelen door de gootsteen of het toilet* - *Ingeleverd bij de apotheek, winkel of milieustraat [Voor Vlaanderen: Ingeleverd bij de winkel, recyclagepark of inzamelpunt klein gevaarlijk afval (kga)]* |
