# Supplementary material for: Assessing tap water awareness: The development of an empirically-based framework
Source: PLoS One. 2021 Oct 29;16(10):e0259233. doi: 10.1371/journal.pone.0259233 (PMC8555835; doi:10.1371/journal.pone.0259233)
Supplement: S2 Appendix — (DOCX) [file pone.0259233.s002.docx]

**Appendix - Results per question**

**Legend: significance: *p*-value**

P-values are given for scores that differ significantly from the average score of the others. Significant differences are marked in gray.

|  | *p*-value |
| --- | --- |
| * | *p* < .05 |
| ** | *p* < .01 |
| *** | *p* < .001 |

**Results Cognition**

| **Dimension** | **No.** | **Total Score** | **Gender**  (n=1001) | **Age** (n=996) | | | | | | |
| --- | --- | --- | --- | --- | --- | --- | --- | --- | --- | --- |
|  |  |  |  | **≤17** | **18 -24** | **25 - 34** | **35 - 44** | **45 -54** | **55 -64** | **65≥** |
| **Cognition** | **I.1** | 63.9 | ♀ 66.5 ♂ 60.9 | 66.7 | 62.4 | 62.9 | 57.4 | 67.5 | 64.0 | 68.4 |
|  | **I.2** | 40.5 | ♀ 40.6 ♂ 40.4 | 30.0 | 42.7 | 37.1 | 43.2 | 40.8 | 37.0 | 44.6 |
|  | **I.3** | 29.4 | ♀ 33.5 ♂ 24.7*  *t =* 3.08 (s) | 16.7 | 30.8 | 28.2 | 33.5*  *t =* 2.85 (vs) | 25.5 | 28.0 | 32.8 |
|  | **II.1** | 23.5 | ♀ 25.8 ♂ 20.7 | 19.2 | 19.0 | 20.0 | 26.9 | 25.5 | 23.0 | 26.8 |
|  | **II.2** | 44.9 | ♀ 45.6 ♂ 44.1 | 40.8 | 47.0 | 41.8 | 37.9 | 48.7 | 48.8 | 46.9 |
|  | **III.1** | 70.1 | ♀ 89.3 ♂ 47.6***  *t =* 15.48 (m) | 65.5 | 74.8 | 61.8 | 63.6 | 73.1 | 75.5*  *t =* 2.79 (vs) | 74.1*  *t =* 2.59 (vs) |
|  | **III.2** | 26.6 | ♀ 24.7 ♂ 29.0 | 23.3 | 35.9 | 26.5 | 23.2 | 22.9 | 28.5 | 24.9 |
|  | **III.3** | 52.1 | ♀ 54.2 ♂ 49.8 | 57.8 | 51.9 | 49.6 | 51.1 | 53.5 | 48.9 | 57.0 |
|  | **III.4** | 56.8 | ♀ 58.3 ♂ 54.8 | 58.6 | 54.3 | 55.9 | 54.5 | 60.3 | 55.5 | 59.2 |
|  | **III.5** | 57.8 | ♀ 60.1 ♂ 55.1 | 58.3 | 58.5 | 59.9 | 52.7 | 56.2 | 56.5 | 62.7 |

| **Dimension** | **No.** | **Total score** | **Education** (n=1000) | | | **Customer perspective** (n=999) | | | |
| --- | --- | --- | --- | --- | --- | --- | --- | --- | --- |
|  |  |  | **Low** | **Middle** | **High** | **Quality & health concerned** | **Aware & commited** | **Egalitarian & soldiary** | **Down to earth & confident** |
| **Cognition** | **I.1** | 63.9 | 54.6** *t =* -3.41 (s) | 62.5 | 71.4*** *t =* 4.16 (s) | 58.1 | 69.8* *t =* 2.89 (vs) | 64.1 | 59.2 |
|  | **I.2** | 40.5 | 36.3 | 37.0 | 46.93* *t =* 3.02 (s) | 33.3 | 46.3 | 36.6 | 40.8 |
|  | **I.3** | 29.4 | 31.3 | 28.1 | 29.6 | 27.1 | 32.6 | 28.5 | 27.5 |
|  | **II.1** | 23.5 | 19.0 | 24.8 | 25.1 | 27.7 | 24.0 | 22.7 | 21.8 |
|  | **II.2** | 44.9 | 40.9 | 44.2 | 48.2 | 37.6 | 46.4 | 43.8 | 47.8 |
|  | **III.1** | 70.1 | 68.5 | 68.6 | 72.8 | 62.2 | 71.3 | 71.5 | 71.1 |
|  | **III.2** | 26.6 | 15.4*** *t =* -5.20 (s) | 25.3 | 35.3*** *t =* 5.05 (s) | 23.3 | 31.4 | 22.5 | 26.8 |
|  | **III.3** | 52.1 | 50.4 | 51.5 | 53.8 | 46.7 | 52.5 | 49.4 | 57.2 |
|  | **III.4** | 56.8 | 55.9 | 55.7 | 58.4 | 58.3 | 60.2 | 51.4 | 57.6 |
|  | **III.5** | 57.8 | 51.3* *t =* -3.02 (vs) | 59.4 | 60.4 | 55.0 | 54.6 | 55.5 | 65.7*** *t =* 3.93 (s) |

**Results Affection**

| **Dimension** | **No.** | **Total Score** | **Gender**  (n=1001) | **Age** (n=996) | | | | | | |
| --- | --- | --- | --- | --- | --- | --- | --- | --- | --- | --- |
|  |  |  |  | **≤17** | **18 -24** | **25 - 34** | **35 - 44** | **45 -54** | **55 -64** | **65≥** |
| **Affection** | **IV.1** | 83.9 | ♀ 83.3 ♂ 84.7 | 82.5 | 87.6 | 82.4 | 82.2 | 81.7 | 84.6 | 86.2 |
|  | **IV.2** | 16.9 | ♀ 17.7 ♂ 16.0 | 22.5 | 17.9 | 16.2 | 17.4 | 18.5 | 15.6 | 15.3 |
|  | **IV.3** | 37.9 | ♀ 42.4 ♂ 32.6***  *t =* 4.38 (s) | 42.5 | 32.7 | 36.9 | 36.3 | 46.0 | 37.6 | 35.5 |
|  | **V.1** | 67.9 | ♀ 69.4 ♂ 66.2 | 68.3 | 67.9 | 66.3 | 64.4 | 70.4 | 70.8 | 67.4 |
|  | **V.2** | 58.3 | ♀ 64.2  ♂ 51.4***  *t =* 5.67 (s) | 68.3 | 57.3 | 57.1 | 59.4 | 62.4 | 60.5 | 50.8 |
|  | **V.3** | 47.1 | ♀ 52.1 ♂ 41.0*** *t =* - 3.452 (s) | 50.8 | 42.5 | 43.4 | 42.1 | 56.5 | 51.4 | 43.5 |
|  | **VI.1** | 58.3 | ♀ 62.0 ♂ 53.7 ***  *t =* 4.76 (s) | 65.0 | 55.3 | 59.3 | 54.4 | 64.0 | 58.9 | 55.7 |
|  | **VI.2** | 58.2 | ♀ 63.5 ♂ 51.7***  *t =* 5.20 (s) | 58.3 | 58.1 | 55.6 | 58.1 | 61.8 | 57.8 | 57.3*  *t =* 2.58 (vs) |

| **Dimension** | **No.** | **Total score** | **Education** (n=1000) | | | **Customer perspective** (n=999) | | | |
| --- | --- | --- | --- | --- | --- | --- | --- | --- | --- |
|  |  |  | **Low** | **Middle** | **High** | **Quality & health concerned** | **Aware & commited** | **Egalitarian & soldiary** | **Down to earth & confident** |
| **Affection** | **IV.1** | 83.9 | 79.4*** *t =* -4.03 (s) | 84.6 | 86.3** *t =* 3.28 (s) | 71.3*** *t =* -5.86 (s) | 87.2*** *t =* 5.09 | 81.6 | 88.4*** *t =* 6.10 (s) |
|  | **IV.2** | 16.9 | 15.3 | 16.6 | 18.3 | 20.5 | 18.4 | 18.0 | 12.2*** *t =* -4.64 (s) |
|  | **IV.3** | 37.9 | 35.5 | 37.0 | 40.4 | 50.0*** *t =* 4.19 (s) | 42.8 | 42.3 | 21.4*** *t =* -10.12 (m) |
|  | **V.1** | 67.9 | 66.9 | 68.6 | 67.9 | 66.3 | 74.9*** *t =* 7.033 (s) | 69.7 | 58.0*** *t =* -7.60 (s) |
|  | **V.2** | 58.3 | 62.3 | 58.4 | 55.7 | 62.8 | 62.5 | 63.6* *t =* 2.63 (vs) | 45.5*** *t =* -6.62 (s) |
|  | **V.3** | 47.1 | 46.5 | 45.4 | 49.0 | 55.2** *t =* 3.22 (s) | 53.4*** *t =* 3.70 (s) | 52.0* *t =* 2.70 (s) | 29.9*** *t =* -8.84 (s) |
|  | **VI.1** | 58.3 | 57.7 | 56.3 | 60.7 | 67.1*** *t =* 4.60 (s) | 65.2*** *t =* 4.64 (s) | 58.0 | 45.6*** *t =* -9.03 (s) |
|  | **VI.2** | 58.2 | 57.9 | 55.7 | 60.7 | 65.1* *t =* 2.39 (vs) | 66.6*** *t =* 4.58 (s) | 59.0 | 43.4*** *t =* -7.66 (s) |

**Results Behavior**

| **Dimension** | **No.** | **Total Score** | **Gender**  (n=1001) | **Age** (n=996) | | | | | | |
| --- | --- | --- | --- | --- | --- | --- | --- | --- | --- | --- |
|  |  |  |  | **≤17** | **18 -24** | **25 - 34** | **35 - 44** | **45 -54** | **55 -64** | **65≥** |
| **Behavior** | **VII.1** | 11.4 | ♀ 11.9 ♂ 10.9 | 16.7 | 12.8 | 11.2 | 13.5 | 13.4 | 7.5 | 10.3 |
|  | **VII.2** | 65.5 | ♀ 66.0 ♂ 64.8 | 65.6 | 65.2 | 63.5 | 62.0 | 68.0 | 67.2 | 67.0 |
|  | **VIII.1** | 89.5 | ♀ 90.2 ♂ 88.8 | 92.5 | 88.7 | 91.6 | 88.4 | 87.9 | 88.9 | 91.0 |
|  | **VIII.2** | 51.9 | ♀ 58.4 ♂ 44.3***  *t =* 7.00 (s) | 51.7 | 44.7 | 50.7 | 50.5 | 54.3 | 52.9 | 55.8 |
|  | **IX.1** | 71.5 | ♀ 70.2 ♂ 73.3*** *t =* 24.23 (L) | 66.1 | 60.2 | 65.0 | 78.2 | 76.7 | 70.3 | 76.7 |
|  | **IX.2** | 72.0 | ♀ 71.7 ♂ 72.9 | 62.5 | 64.7* *t =* -2.07 (vs) | 70.7 | 75.2 | 73.1 | 72.6 | 76.3* *t =* 2.38 (vs) |

| **Dimensie** | **No.** | **Total score** | **Education** (n=1000) | | | **Customer perspective** (n=999) | | | |
| --- | --- | --- | --- | --- | --- | --- | --- | --- | --- |
|  |  |  | **Low** | **Middle** | **High** | **Quality & health concerned** | **Aware & commited** | **Egalitarian & solidary** | **Down to earth & confident** |
| **Behavior** | **VII.1** | 11.4 | 6.3** *t =* - 3.44 (s) | 10.1 | 16.2** *t =* 3.56 (s) | 24.8*** *t =* 3.87 (s) | 12.8 | 8.5* *t =* -2.75 (vs) | 6.4*** *t =* -4.13 (s) |
|  | **VII.2** | 65.5 | 61.5 | 65.0 | 68.5 | 53.0*** *t =* -3.73 (s) | 71.5*** *t =* 4.15 (s) | 63.8 | 65.7 |
|  | **VIII.1** | 89.5 | 87.1 | 89.6 | 91.0 | 84.5 | 92.5** *t =* 3.40 (s) | 90.6 | 87.2 |
|  | **VIII.2** | 51.9 | 52.7 | 53.9 | 49.3 | 56.8 | 53.0 | 53.5 | 46.5** *t =* -3.34 (s) |
|  | **IX.1** | 71.5 | 79.8*** *t =* 5.03 (s) | 72.4 | 64.5*** *t =* -5.22 (s) | 66.1 | 74.1 | 75.5 | 66.6 |
|  | **IX.2** | 72.0 | 78.5*** *t =* 3.55 | 70.2 | 69.8 | 68.0 | 73.7 | 76.3** *t =* 2.60 (vs) | 67.4* *t =* -2.362 (vs) |
