# Supplementary material for: Assessing tap water awareness: The development of an empirically-based framework
Source: PLoS One. 2021 Oct 29;16(10):e0259233. doi: 10.1371/journal.pone.0259233 (PMC8555835; doi:10.1371/journal.pone.0259233)
Supplement: S3 Appendix — (DOCX) [file pone.0259233.s003.docx]

**Appendix – Additional TWA Scores**

|  | **Total score** | | **Income** | | | | | **Family size** | | | | | | | | |
| --- | --- | --- | --- | --- | --- | --- | --- | --- | --- | --- | --- | --- | --- | --- | --- | --- |
|  |  |  | **< €15.000** | | **€15,000 - €30,000** | | **€30,000 - €60,000** | **€60,000 >** | | **1** | | **2** | | **3** | **4** | **5<** |
|  | | | | | | | | | | | | | | | | |
| **Total** | 53.6 | 50.1**  *t =* - 3.21 (s) | | 54.2 | | 54.5 | | 54.1 | 53.6 | | 55.2***  t = 4.29 (s) | | 51.5 | | 52.5 | 51.2 |
|  | | | | | | | | | | | | | | | | |
| **Cognition** | 44.9 | | 41.4 | | 43.8 | | 47.7***  *t =* 3.56 (s) | 46.7 | | 45.9 | | 45.9 | | 43.3 | 43.5 | 44.0 |
| **Affection** | 56.9 | | 56.0 | | 59.0 | | 55.8 | 56.1 | | 56.6 | | 58.0 | | 55.3 | 56.8 | 55.5 |
| **Behaviour** | 60.3 | | 53.9**  *t =* - 3.79 (s) | | 61.5 | | 61.7 | 60.9 | | 59.7 | | 63.7***  *t =* 5.28 (s) | | 57.2 | 58.5 | 54.9 |
|  | | | | | | | | | | | | | | | | |
| **Quality** | 48.2 | | 45.2 | | 48.1 | | 49.5 | 48.4 | | 49.6 | | 48.3 | | 45.9 | 48.3 | 48.4 |
| **Quantity** | 54.8 | | 53.5 | | 56.5 | | 54.1 | 54.5 | | 53.9 | | 55.8 | | 52.9 | 55.8 | 53.2 |
| **System** | 57.9 | | 52.5**  *t =* - 3.38 (s) | | 58.6 | | 59.9 | 59.9 | | 57.4 | | 61.8***  *t =* 6.13 (s) | | 56.2 | 54.0 | 52.4 |

| **Dimension** | **Component** | **Income** | | | | **Family size** | | | | |
| --- | --- | --- | --- | --- | --- | --- | --- | --- | --- | --- |
|  |  | **< €15.000** | **€15,000 - €30,000** | **€30,000 - €60,000** | **€60,000 >** | **1** | **2** | **3** | **4** | **5<** |
|  |  |  |  |  |  |  |  |  |  |  |
| **Cognition** | **(I) Water quality comprehension** | 37.7 | 40.6 | 42.4 | 39.2 | 41.4 | 40.8 | 39.0 | 39.6 | 42.2 |
|  | **(II) Water consumption knowledge** | 32.2 | 34.2 | 36.1 | 33.0 | 37.4 | 34.2 | 31.1 | 34.9 | 31.3 |
|  | **(III) Water system understanding** | 47.5 | 49.9 | 55.8**  t = 3.40 (s) | 57.0*  *t =* 2.92 (vs) | 52.1 | 54.0 | 51.2 | 49.4 | 50.7 |
| **Affection** | **(IV) Water quality perception** | 53.8 | 55.9 | 56.3 | 55.6 | 56.0 | 55.5 | 54.8 | 56.2 | 56.0 |
|  | **(V) Caring for water** | 58.8 | 62.0 | 54.6 | 54.7 | 56.9 | 59.3 | 55.4 | 58.0 | 56.3 |
|  | **(VI) Sense of responsibility** | 56.3 | 60.7 | 56.3 | 59.1 | 57.4 | 61.1 | 56.0 | 56.5 | 53.5 |
| **Behaviour** | **(VII) Quality-driven behaviour** | 39.1 | 43.1 | 45.6 | 46.0 | 47.4 | 44.3 | 38.5 | 44.2 | 43.4 |
|  | **(VIII) Curtailment & efficiency behaviour** | 66.8 | 70.6 | 71.2 | 75.6 | 66.0***  *t =* - 3.27 (s) | 72.0 | 70.9 | 73.5 | 70.7 |
|  | **(IX) Tap water source protection** | 58.4 | 72.6 | 70.2 | 64.1 | 67.1 | 76.4**  *t =* 3.58 (s) | 65.0 | 60.8 | 54.7 |
