# Supplementary material for: Assessing tap water awareness: The development of an empirically-based framework
Source: PLoS One. 2021 Oct 29;16(10):e0259233. doi: 10.1371/journal.pone.0259233 (PMC8555835; doi:10.1371/journal.pone.0259233)
Supplement: S4 Appendix — (DOCX) [file pone.0259233.s004.docx]

**Appendix – PCA and Multiple regression**

**Principle Component Analysis**

A Principle Component Analysis (PCA) was conducted on the 24 items (i.e., the frameworks’ questions listed in Table 3) with orthogonal rotation (varimax)^[[1]](#footnote-1)^. An initial analysis was run to obtain eigenvalues for each component in the data. Eight components had eigenvalues over Kaiser’s criterion of 1 and in combination explained 51.11% of the variance. The scree plot was slightly ambiguous and showed inflexions that would justify retaining components 1 and 2 (this combination explains 21.2% of the variance).

**Multiple regression analysis**

The multiple regression analysis was applied with the aim of exploring which questions are most predictive for the overall score. As mentioned in the main text of the paper, the following questions turned out to be the most predictive for the overall TWA score: questions on (i) the disposal of leftover medicine (question IX.1); (ii) the consumption of bottled water (question VII.2); (iii) the knowledge that there is no chlorinate in the Dutch drinking water (question I.2); (iv) the knowledge that quality requirements are more stringent for tap water than for bottled water (question I.3), and (v) the knowledge on the origin of tap water (question III.1).

On the other end, and not mentioned in the main text of the paper, question IV.2 appeared to be the least predictive since most respondents replied in the same manner, i.e., they took drinking water services for granted. Accordingly, the intention to save water (question V.1) was the second least predictive question (i.e., most respondents wanted to save water). Most respondents also indicated they turned off the tap when brushing their teeth (question VIII.1), knew that tap water contained anthropogenic substances (question I.1) and know that (Dutch) water utilities are responsible for the water quality up to the point where it enters their home (question III.4).

Table 1 Multiple regression analysis results. The weighting for each question can be found in S1.

|  | **Questions / statements** | **B** | **SE B** | | **β** |
| --- | --- | --- | --- | --- | --- |
| No. | Constant | 0.010 | .077 | |  |
| **IX.1** | In the past 24 months, how did you dispose of your old medicines? | 1.059 | 0.006 | | 0.224* |
| **VII.2** | How often do you drink bottled non-sparkling water at home? | 0.999 | 0.005 | | 0.211* |
| **I.2** | To your knowledge, is chlorine added to your tap water? | 0.993 | 0.006 | | 0.183* |
| **I.3** | Are the quality requirements stricter for tap water or bottled water? | 0.999 | 0.006 | | 0.171* |
| **III.1** | What is the source of your tap water? | 0.984 | 0.006 | | 0.169* |
| **III.2** | What is the name of your drinking water utility? | 0.989 | 0.006 | | 0.164* |
| **VI.1** | How safe do you perceive tap water in the Netherlands? | 0.995 | 0.007 | | 0.150* |
| **III.5** | What is the price for 1,000 litres of tap water, excluding taxes? | 0.990 | 0.007 | | 0.143* |
| **II.2** | Estimate how much water, in litres, a conventional shower head uses per minute? | 0.997 | 0.008 | | 0.135* |
| **IV.3** | How often do you think about the quality of your tap water? | 1.010 | 0.010 | | 0.135* |
| **VI.2** | I feel a personal responsibility for protecting the quality of water in rivers, lakes, ditches and subsurface | 0.999 | 0.009 | | 0.134* |
| **V.2** | Every single day I experience 24 hours running tap water as special | 0.992 | 0.009 | | 0.133* |
| **III.3** | Which responsibilities do you think belong to the tasks of your drinking water utility? | 0.995 | 0.008 | | 0.128* |
| **IX.2** | In the past 24 months, how did you dispose of products such as old or used white spirit, stripper, brush softener or old weed killer? | 0.982 | 0.010 | | 0.123* |
| **V.3** | How often do you think about your water consumption? | 0.988 | 0.010 | | 0.123* |
| **II.1** | Estimate the average daily water consumption of one person in the Netherlands? | 0.991 | 0.008 | | 0.121* |
| **VIII.2** | Which of the following water efficient appliances have you installed in your home? | 0.984 | 0.009 | | 0.119* |
| **VII.1** | In the past 24 months, have you ever actively looked for information on the quality and safety of Dutch tap water? | 0.994 | 0.009 | | 0.118* |
| **VI.1** | I sometimes think about the origin of my tap water | 1.006 | 0.012 | | 0.104* |
| **III.4** | Drinking water utilities are responsible for the quality of tap water? (up to the pumping station; the water meter; the tap) | 0.979 | 0.011 | | 0.091* |
| **I.1** | My tap water contains… (none; a small quantity; or a large quantity of anthropogenic substances) | 1.006 | 0.012 | | 0.091* |
| **VIII.4** | What do you do with the tap while tooth brushing? | 0.993 | 0.012 | | 0.087* |
| **V.1** | I would like to save (more) tap water at home | 0.986 | 0.014 | | 0.082* |
| **IV.2** | In my view, clean tap water is something obvious | 0.996 | 0.014 | | 0.079* |
|  | R^2^ = .999 and **p* < .001 |  |  |  | |

1. The kaiser-Meyer-Olkin measure verified the sampling adequacy for the analysis, KMO = .74, which is well above the acceptable limit of .5, Barlett’s test of sphericity X2 (276) = 2581,785, p < .001, indicated that correlations between items were sufficiently large for the PCA. [↑](#footnote-ref-1)
